# Dietary and Nutritional Strategies to Prevent Uremic Toxin Formation and Slow the Progression of Diabetic Kidney Disease

**DOI:** 10.3390/jcm14134701

**Published:** 2025-07-03

**Authors:** Karolina Kędzierska-Kapuza, Anna Grudniewska, Anna Durma, Robert Małecki, Edward Franek, Małgorzata Szczuko

**Affiliations:** 1 Department of Internal Medicine, Endocrinology and Diabetology, National Medical Institute of the Ministry of Interior and Administration in Warsaw, 137 Wołoska Street, 02-507 Warsaw, Poland; edward.franek@pimmswia.gov.pl; 2 Lux Med Medical Centre in Warsaw, 49 Komitetu Obrony Robotników Street, 02-146 Warsaw, Poland; grudniewska.anna@gmail.com; 3 Department of Diabetology and Internal Medicine, Medical University in Warsaw, Żwirki Wigury 61 Street, 02-097 Warsaw, Poland; a__owczarczyk@wp.pl; 4 Department of Nephrology, Miedzyleski Specialist Hospital in Warsaw, Bursztynowa 2 Street, 04-749 Warsaw, Poland; robert.malecki@aol.pl; 5 Department of Bromatology and Nutritional Diagnostics, Pomeranian Medical University in Szczecin, 71-460 Szczecin, Poland

**Keywords:** type 2 diabetes, DKD, CKD, gut–kidney axis, gut microbiota, dysbiosis, nephroprotection, uremic toxins, resistant starch

## Abstract

**Background:** Type 2 diabetes (T2D) is the leading cause of chronic kidney disease (CKD), responsible for approximately 60% of cases. Diabetic kidney disease (DKD) affects 20–50% of individuals with diabetes, with diabetes-related ESKD cases rising steadily worldwide from 22.1% in 2000 to 31.3% in 2015. **Methods:** This review examines the literature published up to 25 February 2025, using a systematic search in PubMed and Scopus. Keywords included uremic toxins and diabetic kidney disease and/or gut microbiota, or dysbiosis or gut–kidney axis. Studies were independently assessed by a minimum of three authors, with discrepancies resolved through consensus. **Results:** Gut microbiota dysbiosis is a key driver of DKD progression, making the gut–kidney axis a promising therapeutic target. A “nuts and fruits” dietary pattern reduces the DKD risk by 43.3%, while an animal protein intake lowers the diabetic peripheral neuropathy risk by 42.8%. High-fiber diets and supplements like resistant starch may reduce uremic toxins through microbiota modulation. **Conclusions:** Microbiota-targeted interventions, including probiotics, synbiotic, and dietary modifications, show potential in reducing uremic toxin production and inflammation, though DKD-specific evidence remains limited. *Lactobacillus* and *Bifidobacterium* strains may help lower urea and creatinine levels, but outcomes vary by disease stage. Further research is needed to confirm the efficacy of dietary and probiotic approaches in DKD management.

## 1. Introduction

Epidemiological data indicate that chronic kidney disease (CKD) affects approximately 11% of adults, which translates to over 800 million people worldwide [1]. Diabetic kidney disease (DKD) remains one of the leading causes of end-stage kidney disease (ESKD) worldwide. However, epidemiological data suggest that the contribution of specific etiologies to ESKD varies by region. For example, according to the ERA-EDTA Registry analysis by Jager et al. [1], hypertensive nephrosclerosis (nephroangiosclerosis) accounts for an incidence of ESKD that is comparable to or slightly higher than DKD in some European populations. This highlights the importance of considering regional and demographic variability in the interpretation of ESKD trends. Nonetheless, due to the global rise in type 2 diabetes prevalence, DKD continues to represent a major and growing public health burden across diverse populations. The number of people with diabetes is expected to increase from 537 to 783 million over the next 24 years, leading to a rise in the global prevalence of DKD, which affects 20% to 50% of people with diabetes [2]. Type 2 diabetes (T2DM) accounts for 90% of all diabetes cases worldwide; consequently, the majority of individuals who develop DKD are affected by this type of diabetes [3,4]. Reflecting this trend, the global proportion of ESKD attributable to diabetes rose steadily from 22.1% in 2000 to 31.3% in 2015. DKD is the most common cause of ESKD, which requires renal replacement therapy. This condition is associated with high mortality and a significant socio-economic burden, posing a challenge to public health worldwide. Therefore, new alternative strategies for protecting against DKD and slowing the progression of DKD in the course of T2DM are urgently needed [5]. Diabetes contributes to changes in the glomeruli, which increase the permeability of the small blood vessels in the glomeruli, causing albumin to pass from the blood into the urine. The metabolic control of diabetes is thus crucial and includes the treatment of hyperglycemia, dyslipidemia, hypertension, and obesity.

According to KDIGO 2024 recommendations, the management of DKD is now conceptualized around four therapeutic pillars, each supported by strong evidence for renal and cardiovascular protection [6]:

- Renin–Angiotensin–Aldosterone System (RAAS) Inhibition: ACE inhibitors or ARBs are recommended in patients with albuminuria to reduce proteinuria and control blood pressure. A RAAS blockade remains foundational in slowing DKD progression.
- Sodium–glucose co-transporter 2 inhibitors are recommended for all patients with proteinuric CKD, irrespective of their diabetes status, due to their proven nephroprotective and cardioprotective effects.
- Non-steroidal Mineralocorticoid Receptor Antagonist (n-MRA): finerenone, as the only approved agent in this class for DKD, is recommended for patients with T2DM and DKD. It provides an additional renal and cardiovascular benefit beyond RAAS inhibition.
- Glucagon-like peptide-1 receptor agonists are advised in patients with type 2 diabetes and DKD, particularly when glycemic control, weight reduction, or further cardiovascular protection is indicated.

Well-established interventions—most notably the inhibition of the RAAS—have led to consistent improvements in patient outcomes over the past three decades. In the context of T2DM, the most compelling evidence for delaying the progression of DKD stems from trials involving angiotensin receptor blockers (ARBs), particularly the IDNT and RENAAL studies [7,8]. ACE inhibitors have a similar effect on T2DM, as shown in the secondary analysis of the ADVANCE study [9].

A breakthrough in therapy was the addition of sodium–glucose cotransporter 2 (SGLT2) inhibitors to RAASi. In the CEREDENCE study, the nephroprotective efficacy of canagliflozin in diabetic kidney disease was proved; in the DAPA-CKD and the EMPA-Kidney studies, such efficacy was proved for dapagliflozin and empagliflozin, respectively, in both diabetic and non-diabetic kidney disease [10,11,12].

At the same time, the FIDELIO and FIGARO studies showed that treatment with Finerenone (nMRAs) slows down the loss of kidney function, regardless of the possible effect of this drug on the heart [13]. There are also the results of the first nephrological study using glucagon-like peptide-1 receptor agonists (GLP-1 RAs), FLOW, where the nephroprotective effect of semaglutide, administered subcutaneously to patients with DKD, was confirmed [14].

Nephroprotective effects were also observed in a post hoc analysis of a randomized controlled clinical trial of tirzepatide— a recently launched medication intended for the treatment of diabetes and obesity, which activates both glucose-dependent insulinotropic polypeptide and GLP-1 receptors [15].

Thus the emergence of these new groups of drugs has initiated a significant paradigm shift in combating DKD and cardiovascular complications [16,17].

However, despite glycemic and blood pressure control and treatment with RAAS inhibitors, alongside with modern pharmacotherapy with SGLT2 inhibitors, nMRA, or GLP-1RA, there remains a significant residual risk of the further progression of DKD. The rate of eGFR decline with RAAS blockers was estimated at 4–6 mL/min/year; current modern pharmacotherapy slows this decline to around 2 mL/min/year, but the optimal physiological annual decline is approximately 0.7–0.9 mL/min/year [18]. The rapid progression of DKD is influenced by several known molecular mechanisms such as changes in cellular metabolism due to an increased glucose uptake, renal hypoxia, and the production of reactive oxygen species with subsequent tissue inflammation and fibrosis, changes in renal hemodynamics, and, until recently, not associated with a worsening renal prognosis, gut microbiota dysbiosis [19]. Unraveling how the gut microbiota drives kidney damage in diabetes is not only critical for DKD treatment, but may also lead to novel, targeted therapeutic approaches.

### 1.1. Uremic Toxins—The Role of the Gut–Kidney Axis

Uremic toxins are commonly categorized based on their molecular size and water solubility: small, water-soluble molecules such as urea and phosphorus; medium-sized molecules like β2-microglobulin; and protein-bound solutes including indoxyl sulfate (IS), p-cresyl sulfate (pCS), and homocysteine. Their precursors arise from the bacterial fermentation of amino acids—phenylalanine, tyrosine, and tryptophan—which are metabolized into compounds such as p-cresol, phenol, and indole.

The analysis of women with gestational diabetes showed that impaired tryptophan metabolism (the level of the amino acid 5-hydroxytryptophan) is the most important pathway involved in gestational diabetes mellitus (GDM). 5-Hydroxytryptophan is a precursor of serotonin and can increase serotonin levels [20]. Serotonin can regulate insulin secretion from pancreatic β-cells via a protein serotoninylation mechanism. These molecules can effectively predict the transition from GDM to T2DM [21]. As they pass through the intestinal wall and/or the liver, these precursors undergo conjugation, giving rise to circulating uremic retention solutes and toxins—such as p-cresyl sulfate, p-cresyl glucuronide, phenyl sulfate, phenyl glucuronide, indoxyl sulfate, and indoxyl glucuronide. These compounds exert biological effects by promoting inflammation, activating leukocytes, and impairing the endothelial function. Trimethylamine N-oxide (TMAO), produced from choline, phosphatidylcholine, carnitine, and betaine and other intestinal metabolites, such as trimethylamines (TMA) and short-chain fatty acids (SCFA), reach the brain by crossing the blood–brain barrier and are involved in metabolic, vascular, and neurodegenerative disorders in the course of type 2 diabetes [22]. TMAO affects the increase in oxidative stress, microglia activation, and the apoptosis of brain neurons, leading to the development of mental, cognitive, and behavioral disorders among patients [23]. If the significant chronic intestinal dysbiosis and overproduction of toxin precursors occur, it can become a triggering factor for the onset of DKD and, through the spread of toxins and increasing renal overload, increase the risk of ESKD [24]. There is a known association between intestinal inflammation, dysbiosis, and circulating uremic toxins; this connection indicates a gut–kidney axis, particularly in the context of CKD development [25,26]. The production of ROS in the gastrointestinal tract by a diverse cohort of commensal microbes is a regulated process leading to the generation of hydrogen peroxide. Its action has been implicated in the essential signaling required for normal cellular homeostasis and physiological function in preventing the progression of kidney disease [25].

### 1.2. Intestinal Dysbiosis

Alterations in gut microbiota have been consistently linked to declining kidney function [27,28]. As the glomerular filtration rate decreases, the accumulation of uremic toxins and disruptions in acid base homeostasis lead to both quantitative and qualitative shifts in microbial composition [29,30]. These dysbiotic changes are accompanied by increased intestinal permeability, facilitating the translocation of bacteria and their toxic metabolites into the circulation. This translocation contributes to chronic systemic inflammation and oxidative stress, further accelerating renal injury [31,32]. Among the key drivers of dysbiosis in patients with CKD is the breakdown of urea by microbial ureases in the gastrointestinal tract. This enzymatic activity raises intraluminal ammonia levels and increases the intestinal pH, creating an environment conducive to microbial imbalance [26]. A study conducted in 2014 [33] found that 63% of the 19 predominant bacterial genera identified in CKD patients harbored genes encoding urease. An elevated intestinal pH also promotes the expression of tryptophanase, an enzyme that catalyzes the conversion of tryptophan to indole—a precursor of the uremic toxin indoxyl sulfate (IS). Microbial dysbiosis in CKD is further associated with a reduced capacity for the synthesis of beneficial metabolites such as short-chain fatty acids (SCFAs). Wong et al. [34] demonstrated that two microbial families known to produce SCFAs—Prevotellaceae and Lactobacillaceae, especially those involved in butyrate production—were significantly depleted in ESKD patients. Moreover, the diffusion of urea into the intestinal lumen favors the proliferation of urease-producing bacteria, with ammonia and ammonium hydroxide byproducts contributing to epithelial injury and the breakdown of the intestinal barrier. As this barrier deteriorates, endotoxins such as lipopolysaccharides (LPS) derived from Gram-negative bacteria can enter the bloodstream. This triggers innate immune responses through the activation of Toll-like receptor 4 (TLR4) and downstream signaling cascades involving nuclear factor kappa B (NF-κB) and mitogen-activated protein kinases (MAPKs). The result is an amplified inflammatory response characterized by the increased production of cytokines including tumor necrosis factor-alpha (TNF-α), interleukin-1β (IL-1β), interleukin-6 (IL-6), and activator protein 1 (AP-1).

The microbiome features associated with better kidney health were linked to various serum metabolites (e.g., higher levels of indolepropionate and beta-cryptoxanthin; lower levels of imidazole propionate, deoxycholic acid metabolites, and p-cresol glucuronide). Imidazole propionate, deoxycholic acid metabolites, and p-cresol glucuronide were associated with potential reductions in eGFR and/or increases in the albumin-to-creatinine ratio (UACR) over approximately 6 years. Authors concluded that kidney function is a significant correlate of the gut microbiome, while the relationship between kidney damage and the gut microbiome depends on the diabetes status. Gut microbiome metabolites may contribute to the progression of DKD [34].

In a mouse model of diet-induced obesity (DIO), echocardiographic assessments revealed subtle cardiac remodeling. A high-frequency ultrasound (HFUS) of the liver in animals fed a Western-type high-fat diet (WD) demonstrated a progressive increase in both echogenicity and echotextural heterogeneity. Notably, the renal cortex exhibited equal or greater brightness compared to surrounding tissues, and signs of impaired renal perfusion were also observed [35,36] This complex interplay between comorbidities, dysbiosis, and the immune response underscores the role of gut microbiome imbalances in influencing both systemic inflammation and organ dysfunction, particularly in conditions like COVID-19, obesity, and related chronic diseases. The dysregulated microbiome could exacerbate inflammation, impacting vitamin D metabolism, cytokine expression, and kidney function, thereby contributing to the overall pathophysiology of these conditions.

### 1.3. Targeted Supplementation

The impact of various dietary components, including functional foods, on the progression of DKD was presented by Liu, Wang [37], and colleagues. Six dietary patterns were constructed, namely “animal protein,” “whole grains and plant protein,” “nuts and fruits,” “refined grains and vegetables,” “dairy,” and “added sugars,” with the participation of these products in the daily diet amounting to 15.42%, 9.99%, 8.23%, 8.16%, 7.56%, and 7.28%, respectively, which constitutes 56.64% of the total diet composition. It was shown that in patients where the dietary pattern was dominated by the “nuts and fruits” group, the risk of DKD was 43.3% lower compared to patients with the lowest consumption of these products [odds ratio (OR) = 0.567; 95% confidence interval (CI), 0.359–0.894; *p* < 0.001]. Interestingly, in patients with the highest consumption of “animal protein,” the risk of DPN (diabetes peripheral neuropathy) was 42.8% lower compared to patients with the lowest intake of animal proteins in the diet (OR = 0.572; 95% CI, 0.388–0.843; *p* < 0.05). The results of this study suggest that in patients with T2DM, a diet based on nuts and fruits reduces the risk of DKD, while a diet based on animal protein reduces the risk of diabetic peripheral neuropathy [37]. A high-protein diet may exacerbate proteinuria and further deteriorate renal function. Plant proteins are preferred over animal proteins because they reduce the renal burden. This helps to decrease the severity of proteinuria and slow the progression of the disease [38,39]. Impaired gut–kidney axis function in the course of DKD can be modulated by using preparations that modify the composition of the gut microbiota and restore eubiosis—prebiotics, probiotics, and synbiotics. According to the ISAAP (International Scientific Association of Probiotics and Prebiotics) [40], the following can be stated.

Prebiotics—substrates selectively used by microorganisms: dietary fiber including resistant starch and fructooligosaccharides such as inulin and oligofructose.

Probiotics—living organisms which, when administered in appropriate amounts, have a beneficial health effect on the host. These include lactic acid-producing bacteria of the genera *Lactobacillus* (e.g., *L. acidophilus*, *L. casei*, *L. reuteri*, and *L. rhamnosus)* and *Bifidobacterium* (*B. animalis* and *B. breve*), as well as the yeast *Saccharomyces boulardii.*

Synbiotic—a dietary supplement containing a freeze-dried mixture of live bacterial cultures from selected strains, supplemented with fructooligosaccharides.

**Figure 1:**
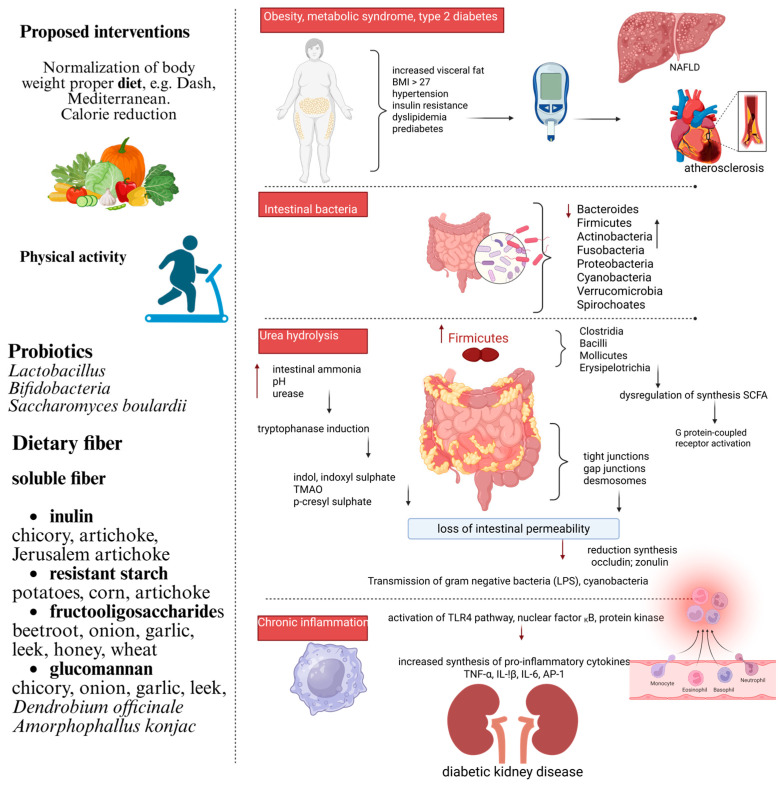
Mechanism of uremic toxin formation during the course CKD and proposed interventions. NAFLD—nonalcoholic fatty liver disease; SCFA—short-chain fatty acids; TMAO—trimethylamine N-oxide; TLR4—Toll-like receptor 4; LPS—lipopolysaccharides; TNF-α—tumor necrosis factor-alpha; IL—interleucin; AP-1—activator protein 1. (Created with BioRender.com; https://app.biorender.com/, accessed on 10 March 2025).

Numerous studies have investigated strategies to reverse dysbiosis and counteract its detrimental effects in patients with chronic kidney disease. A primary focus has been placed on the use of probiotics, prebiotics, and synbiotics, which aim to shift the microbial balance from proteolytic to saccharolytic species, thereby reducing the generation of uremic toxins. The evidence suggests that the administration of these agents positively influences cresol and indole metabolism [41,42,43]. Similarly, Esgalhado et al. [44] observed that resistant starch supplementation for 4 weeks reduced IS levels during hemodialysis. Moreover, the daily intake of dietary fiber (resistant starch) for 6 weeks reduced free IS levels in plasma in hemodialysis patients [45]. Another study found that a high-fiber diet consumed for 3 weeks modulated the gut microbiota in rats with chronic kidney disease, increasing the *Bacteroides* to *Firmicutes* ratio, consequently promoting a reduction in serum IS and p-CS levels [46]. Some active and biologically active plant compounds such as fats, flavonoids, mucus, pigments—anthocyanins, carotenes, and carotenoids—minerals, and vitamins may also have a protective effect. A 2021 study conducted by Salarolli et al. involving hemodialysis patients found that 3-month long curcumin supplementation reduced plasma p-CS levels, likely due to modulation of the gut microbiota [47]. In healthy individuals, four weeks of resveratrol supplementation resulted in reduced levels of trimethylamine N-oxide (TMAO), likely due to the inhibition of TMA-producing gut bacteria [47]. Similarly, in subjects with cardiovascular risk factors, the intake of fermented apple purée favorably altered the gut microbiota composition by increasing *Bifidobacterium* and *Lactobacillus* populations while lowering concentrations of uremic toxins such as TMAO [48]. These findings support the hypothesis that the modulation of the gut microbiota plays an active role in reducing uremic toxin levels and highlight the need for further in-depth research, as this approach may represent a promising therapeutic avenue in DKD. The mechanism of changes in the intestinal microbiota actively involved in the formation of uremic toxins and inflammation is shown in Figure 1. Interventional studies are discussed in more detail later in this article.

## 2. Materials and Methods

**Figure 2:**
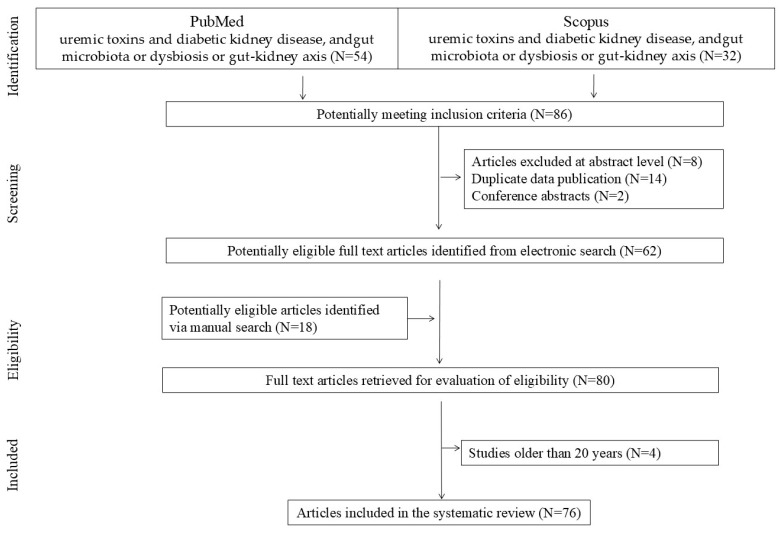
Research flowchart.

This review addresses the aforementioned topics, incorporating relevant the literature published up to 25 February 2025. The studies included in this review were identified through a systematic search of electronic databases and clinical trial registers, conducted in accordance with the PRISMA (Preferred Reporting Items for Systematic Reviews and MetaAnalyses) guidelines. A systematic literature search was performed using the PubMed and Scopus databases. The primary keywords included combinations of the terms: uremic toxins, diabetic kidney disease, and/or gut microbiota, dysbiosis, or gut–kidney axis (PubMed: N = 54; Scopus: N = 32). Duplicate records identified in both databases were excluded (N = 14). Titles and abstracts were initially screened, and studies containing relevant information were subsequently reviewed in full. Finally, articles were added to supplement the content based on merit, i.e., articles identified via a manual search (N = 18). Studies not published in English, as well as letters to the editor and conference abstracts, were excluded in accordance with the selection criteria illustrated in the flow chart (Figure 2). Ultimately, this review includes the most relevant and up-to-date studies published between 2004 and 2025. Data from these studies were extracted independently by two investigators and confirmed by a third investigator.

## 3. Therapeutic Dietary Interventions for Microbiota in DKD

Food components resistant to digestive enzymes positively affect the host by selectively stimulating the growth or activity of bacteria in the colon. This has a documented, beneficial impact on health. Below, we present a review of the literature on microbiota composition interventions used in DKD [49,50,51].

### 3.1. Prebiotics

These substances can be oligo- or polysaccharides that are not digested and reach the intestinal lumen in an unchanged form, where they exert their effects. Prebiotics undergo fermentation in the host’s gastrointestinal tract due to the action of the gut microbiota, producing short-chain fatty acids (SCFA): acetic, propionic, and butyric acid. The best-studied and most commonly used prebiotics are resistant starch (RS), resistant oligosaccharides, fructooligosaccharides, galactooligosaccharides, pectins, inulin, dextrin, glucomannan, and gums [52].

#### 3.1.1. Resistant Starch

Starch, a polysaccharide composed of amylose and amylopectin, is classified as a dietary fiber, with its most beneficial component being resistant starch (RS). This fraction escapes digestion in the small intestine and reaches the colon unchanged, where it undergoes microbial fermentation, leading to the production of short-chain fatty acids (SCFAs). Although there are currently no official dietary recommendations for RS intake, it is generally accepted that consuming at least 15–20 g per day may confer health benefits. However, the available data indicate that the average daily intake of RS in the general population is significantly lower, typically ranging from 3 to 9 g. Foods recognized for their high RS content include green bananas (approximately 6 g per fruit), lentils (3.4 g RS/100 g), muesli (3.3 g RS/100 g), chickpeas (2.6 g RS/100 g), beans (2.0 g RS/100 g), and bran (up to 5 g RS/250 mg). Resistant starch is classified into five main types, each with distinct physicochemical characteristics, as illustrated in Table 1.

RS is a very important substrate for saccharolytic bacteria capable of digesting and fermenting carbohydrates [53]. Most saccharolytic bacteria belong to *Ruminococcus*, *Bifidobacterium*, *Lactobacillus*, or *Eubacterium* spp. [54]. RS increases the populations of *Bifidobacterium* and *Lactobacillus* [55,56,57], which are reduced in diabetic kidney disease (DKD) [58].

In an interventional study by Martínez et al. [59], ten healthy participants were monitored weekly over a 17-week period to assess the individual effects of resistant starch types RS2 and RS4, compared with a control group receiving native starch. A three-week supplementation with RS2 at a daily dose of 33 g resulted in a significant increase in the abundance of *Bifidobacterium adolescentis*, *Eubacterium rectale*, and *Ruminococcus bromii*—microbial species known to ferment resistant starch and produce butyrate. The proliferation of these bacteria in response to RS2 has been well documented [59,60]. In the elderly patient population, RS2 was shown to reduce the concentration of *Proteobacteria* [61], which is significantly increased in DKD [57]. Regarding RS4, Martinez et al. found that a dose of 30 g/day for three weeks was associated with an increase in *Parabacteroides distasonis* and *Bifidobacterium species*, while *R. bromii and Eubacterium* sp. decreased [59]. The observed difference concerned the rate of bacterial growth with the use of different types of resistant starch. While both RS2 and RS4 increased the number of *Bifidobacterium species*, RS2 achieved this change at a slower rate. In men with obesity, a three-week intervention with RS3 at a dose of 25.5 g/day was not associated with changes in *Bifidobacterium* populations, but the number of *E. rectale* and *Ruminococcus* species increased [62]. The findings of Martinez et al. highlight the different effects of each RS subtype on our microbiome [59].

**Figure 3:**
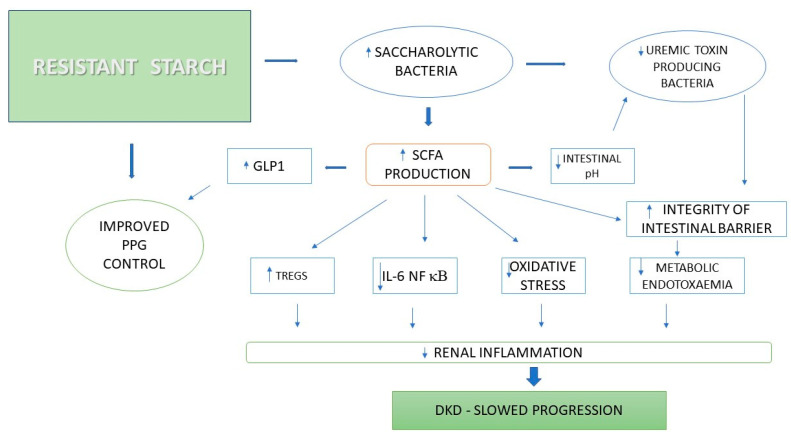
Mechanism of the impact of resistant starch consumption on slowing the progression of diabetic kidney disease. PPG—postprandial glucose, IL-6—interleukin-6, GLP1—glucagon-like peptide 1, NF kB—nuclear factor kappa B, SCFA—short-chain fatty acid, DKD—diabetic kidney disease, TREGS—regulatory T cells, ↑ increase, ↓ decrease.

As shown in Figure 3, the consumption of resistant starch promotes a shift in the gut microbiota towards saccharolytic bacteria and increases the production of metabolites such as short-chain fatty acids (SCFA). SCFA contribute to slowing the progression of DKD by affecting the immune system, modulating the anti-inflammatory response, sealing the intestinal barrier, increasing GLP-1 levels, lowering the intestinal pH, and reducing oxidative stress. Therefore, mechanisms that enhance the integrity of the intestinal barrier are crucial in inhibiting the progression of DKD [63].

One of the most recently published cross-sectional studies examined the impact of the dietary fiber intake in adults with T2D in the United States. Among 6032 participants, 38.4% additionally met the criteria for DKD diagnosis. This study revealed a correlation between a higher DF (dietary fiber) intake and reduced risk of DKD, particularly at the highest level of intake (T3: ≥10.1 g/1000 kcal/day) [64]. Studies on the inclusion of fiber and resistant starch to improve the microbiota in different population groups are presented below in Table 2. At the moment, there are no data in the context of patients with DKD, but it seems that they may have equally good effects.

#### 3.1.2. Fructooligosaccharides

Fructooligosaccharides (FOS) are commonly occurring prebiotics. These are compounds consisting of 1–8 simple sugar molecules of fructose attached to a sucrose molecule. Natural sources of fructooligosaccharides include asparagus, sugar beets, onions, garlic, leeks, wheat, honey, and bananas. FOS are prebiotics, meaning they are non-digestible food ingredients that stimulate the growth of beneficial bacteria in the large intestine and positively impact the host’s health. FOS supplementation may also alleviate the course of CKD [65]. Inulin, oligofructose, and FOS have been extensively studied as prebiotics and have been shown to significantly increase the number of *Bifidobacterium* in feces at relatively low intake levels (5–8 g per day). Very-long-chain inulin extracted from artichoke (*Cynara scolymus*) has shown pronounced prebiotic effects, with good dose tolerance [66]. In another study from 2010, fruit and vegetable “shots” containing inulin from Jerusalem artichoke were given to 66 healthy volunteers, demonstrating its prebiotic potential. After injection, the Bifidobacteria levels were significantly higher compared with a placebo injection. Also, a significant increase in the Lactobacillus/Enterococcus level was observed [67]. Inulin and oligofructose are also found in some other plant-based foods: chicory, artichokes, asparagus, and garlic. In another study which refers to non-diabetic kidney disease, it was observed that fructooligosacharides reduce the serum total and free p-cresyl sulfate, which is uremic toxic [39,68].

#### 3.1.3. Arabic Gum, Polydextrose, and Glucomannan

Notably, a study conducted by Calame W. et al. [69] on healthy volunteers demonstrated that gum arabic (GA) induces a greater increase in *Bifidobacterium* spp. and *Lactobacillus* spp. compared to an equivalent dose of inulin, while causing fewer gastrointestinal side effects such as flatulence and bloating [66,70]. Furthermore, the beneficial effects of GA appear to be mediated through the enhanced production of short-chain fatty acids (SCFAs)—including acetate, propionate, and butyrate—as well as a reduction in oxidative stress. These findings suggest that GA, when used as a prebiotic, may offer therapeutic potential in the management of chronic kidney disease (CKD) [69]. Additional evidence indicates that polydextrose supplementation results in a dose-dependent reduction in *Bacteroides* populations, accompanied by an increase in *Bifidobacterium* and lactic acid bacteria counts [71,72]. Similarly, wheat dextrin has been shown to promote the growth of lactic acid bacteria and *Bifidobacterium*, while simultaneously suppressing *Clostridium perfringens* populations [73].

A study published by Chen et al. [74] showed that diabetic rodents treated with glucomannan (compared to the model group) exhibited lower levels of fasting blood glucose, serum insulin, and HbA1c. The levels of low-density lipoproteins, total cholesterol, triglycerides, and non-esterified fatty acids in the serum were significantly reduced as a result of glucomannan treatment. Additionally, glucomannan administration resulted in significant reductions in serum uric acid, creatinine, and urea levels, as well as decreased glucosuria, ketone bodies, and protein levels in the urine. A histopathological analysis showed that glucomannan treatment normalized the glomerular architecture. Glucomannan derived from the tuberous roots of the plant *Amorphophallus konjac* demonstrated beneficial effects in regulating lipids and glucose. Konjac naturally occurs in Japan and China, where it is used in cuisine to prepare noodles (shirataki), cakes, etc. On the other hand, glucomannan derived from *Dendrobium officinale* more effectively balanced the urea cycle and amino acid metabolism. *D. officinale* Kimura et Migo is a perennial epiphytic herb from the orchid family found in Asia and Oceania [75,76].

### 3.2. Research Directions

Further research is needed on the impact of probiotics and synbiotics on different CKD phenotypes, particularly in DKD (the personalization of microbiota therapy). Randomized studies should include longer intervention periods (>8 weeks) and diverse patient groups to determine the effectiveness of probiotic and dietary therapies (the evaluation of long-term effects of interventions). Research on the role of SCFA, sodium butyrate, and receptor modulation (e.g., GPR41 and GPR43) in kidney protection could open new therapeutic possibilities.

## 4. Conclusions

Dietary interventions, such as a high fiber intake and prebiotics, have demonstrated benefits in reducing uremic toxins, but DKD-specific studies are scarce. But, a higher dietary fiber intake (>10.1 g/1000 kcal/day) reduces the risk of DKD. Probiotics and synbiotics, particularly *Lactobacillus* and *Bifidobacterium* strains, may help lower urea and creatinine levels and improve kidney function, though results are inconsistent and disease-stage-dependent. Microbiota-targeted interventions, including probiotics, synbiotics, and dietary modifications, show promise in modulating these mechanisms, but evidence specific to DKD remains limited. Some natural food components, e.g., glucomannan, gum arabic, inulin, and fructooligosaccharides, modulate the composition of the gut microbiome due to their effects on the intestinal barrier and the translocation of uremic toxins, which slow down the progression of chronic kidney disease.

## Figures and Tables

**Table 1:** Five main types of resistant starch.

RS 1	Physically inaccessible or undigestible resistant starch
	Food sources: Legumes Seeds Wholegrains
RS 2	Resistant starch is inaccessible to enzymes due to starch conformation
	Food sources: Raw potato Unripe bananas High-Amylose Maize Starch (HAMS)
RS 3	Resistant starch is created through the process of retrogradation when starch becomes less soluble after being heated and dissolved in water and then cooled
	Food sources: Heated and then cooled rice, pasta, potatoes, bread
RS 4	Resistant starch is created by the chemical modification of starch molecules and resists digestion
	Food sources: Food in which the modified starches have been used (breads, cakes)
RS 5	Starches that are complexed with lipids
	Food sources: Food with high amylose content (e.g., peas, maize, potatoes, wheat)

**Table 2:** The studies on the influence of resistant starch in different groups and fiber in CKD on changes in the microbiome.

Study	Substance	Subjects and Duration of Supplementation	Duration of Study Results
Martinez et al., 2010 [59]	RS 2 or RS 4 about 30 g RS/day	13 healthy adults 17 weeks Analysis of fecal microbiota by PCR-DGGE	RS2 and RS4 show functional differences in the effect on human microbiota RS2—*Ruminococcus bromii* and *Eubacterium rectale* RS4—*Bifidobacterium adolescentis* and *Parabacteroides distasonis*
Baxter et al., 2019 [60]	RPS about 31 g/day (70%RS2) RMS about 22 g/day (50%RS2) Inulin 20 g/day Starch 40 g/day	2 weeks 172 healthy adults N = 39 N = 43 N = 41 N = 49	RPS resulted in the greatest increase in total SCFA, including butyrate RMS and inulin induced distinct changes in fecal communities but did not generate significant increases in fecal butyrate levels
Venkataraman et al., 2016 [61]	RS (50%RS2)	10 days 20 healthy young adults	increased abundance of the butyrogenic microorganism *Eubacterium rectale*
Alfa et al., 2018 [62]	RS2	12 weeks 42 elderly patient	increased numbers of *Bifidobacterium*
Walker et al., 2011 [63]	RS	10 weeks 12 overweight men	increased in the number of *Eubacterium rectale*
Xin-Hua Jia et al., 2024 [65]	Dietary fiber intake	2316 patients with DKD	increased dietary fiber intake is associated with a reduced incidence of DKD

## References

[1] Jager K.J., Kovesdy C., Langham R., Rosenberg M., Jha V., Zoccali C. (2019). A single number for advocacy and communication-worldwide more than 850 million individuals have kidney diseases. Kidney Int..

[2] Hoogeveen E.K. (2022). The epidemiology of diabetic kidney disease. Kidney Dial..

[3] Sun H., Saeedi P., Karuranga S., Pinkepank M., Ogurtsova K., Duncan B.B., Stein C., Basit A., Chan J.C., Mbanya J.C. (2022). IDF diabetes atlas: Global, regional and country-level diabetes prevalence estimates for 2021 and projections for 2045. Diabetes Res. Clin. Pract..

[4] Koye D.N., Shaw J.E., Reid C.M., Atkins R.C., Reutens A.T., Magliano D.J. (2017). Incidence of chronic kidney disease among people with diabetes: A systematic review of observational studies. Diabet. Med..

[5] Cheng H.T., Xu X., Lim P.S., Hung K.Y. (2021). Worldwide epidemiology of diabetes-related end-stage renal disease, 2000–2015. Diabetes Care.

[6] Levin A., Ahmed S.B., Carrero J.J., Foster B., Francis A., Hall R.K., Herrington W.G., Hill G., Inker L.A., Kazancıoğlu R. (2024). KDIGO 2024 clinical practice guideline for the management of diabetes in chronic kidney disease. Kidney Int..

[7] Lewis E.J., Hunsicker L.G., Clarke W.R., Berl T., Pohl M.A., Lewis J.B., Ritz E., Atkins R.C., Rohde R., Raz I. (2001). Renoprotective effect of the angiotensin-receptor antagonist irbesartan in patients with nephropathy due to type 2 diabetes. N. Engl. J. Med..

[8] Brenner B.M., Cooper M.E., De Zeeuw D., Keane W.F., Mitch W.E., Parving H.-H., Remuzzi G., Snapinn S.M., Zhang Z., Shahinfar S. (2001). Effects of losartan on renal and cardiovascular outcomes in patients with type 2 diabetes and nephropathy. N. Engl. J. Med..

[9] Patel A. (2007). ADVANCE collaborative group. Effects of a fixed combination of perindopril and indapamide on macrovascular and microvascular outcomes in patients with type 2 diabetes mellitus (the ADVANCE trial): A randomised controlled trial. Lancet.

[10] Neal B., Perkovic V., Mahaffey K.W., de Zeeuw D., Fulcher G., Erondu N., Shaw W., Law G., Desai M., Matthews D.R. (2017). Canagliflozin and cardiovascular and renal events in type 2 diabetes. N. Engl. J. Med..

[11] Heerspink H.J.L., Stefánsson B.V., Correa-Rotter R., Chertow G.M., Greene T., Hou F.F., Mann J.F.E., Mcmurray J.J.V., Lindberg M., Rossing P. (2020). DAPA-CKD trial committees and investigators. Dapagliflozin in patients with chronic kidney disease. N. Engl. J. Med..

[12] Herrington W.G., Staplin N., Wanner C., Green J.B., Hauske S.J., Emberson J.R., Preiss D., Judge P., Mayne K.J., The EMPA-KIDNEY Collaborative Group (2023). Empagliflozin in patients with chronic kidney disease. N. Engl. J. Med..

[13] Bakris G.L., Agarwal R., Anker S.D., Pitt B., Ruilope L.M., Rossing P., Kolkhof P., Nowack C., Schloemer P., Joseph A. (2020). Effect of finerenone on chronic kidney disease outcomes in type 2 diabetes. N. Engl. J. Med..

[14] Perkovic V., Tuttle K.R., Rossing P., Mahaffey K.W., Mann J.F.E., Bakris G., Baeres F.M.M., Idorn T., Bosch-Traberg H., Lausvig N.L. (2024). Effects of semaglutide on chronic kidney disease in patients with type 2 diabetes. N. Engl. J. Med..

[15] Bosch C.C.S., Soler M.J., Ortiz A., Fernandez-Fernandez B. (2022). Tirzepatide and prevention of chronic kidney disease. Clin. Kidney J..

[16] Naaman S.C., Bakris G.L. (2023). Diabetic nephropathy: Update on pillars of therapy slowing progression. Diabetes Care.

[17] Tuttle K. (2022). Kidney disease: Improving global outcomes diabetes working group. KDIGO 2022 clinical practice guideline for diabetes management in chronic kidney disease. Kidney Int..

[18] Gao Q., Tan N.C., Lee M.L., Hsu W., Choo J. (2023). Comparative effectiveness of first-line antihypertensive drug classes on the maintenance of estimated glomerular filtration rate (egfr) in real world primary care. Sci. Rep..

[19] Watanabe K., Sato E., Mishima E., Miyazaki M., Tanaka T. (2023). What’s new in the molecular mechanisms of diabetic kidney disease: Recent advances. Int. J. Mol. Sci..

[20] Paulmann N., Grohmann M., Voigt J.-P., Bert B., Vowinckel J., Bader M., Skelin M., Jevšek M., Fink H., Rupnik M. (2009). Intracellular serotonin modulates insulin secretion from pancreatic beta-cells by protein serotonylation. PLoS Biol..

[21] Allalou A., Nalla A., Prentice K.J., Liu Y., Zhang M., Dai F.F., Ning X., Osborne L.R., Cox B.J., Gunderson E.P. (2016). A predictive metabolic signature for the transition from gestational diabetes mellitus to type 2 diabetes. Diabetes.

[22] López-Tenorio I.I., Aguilar-Villegas Ó.R., Espinoza-Palacios Y., Segura-Real L., Peña-Aparicio B., Amedei A., Aguirre-García M.M. (2024). Primary prevention strategy for non-communicable diseases (ncds) and their risk factors: The role of intestinal microbiota. Biomedicines.

[23] Mudimela S., Vishwanath N.K., Pillai A., Morales R., Marrelli S.P., Barichello T., Giridharan V.V. (2022). Clinical significance and potential role of trimethylamine n-oxide in neurological and neuropsychiatric disorders. Drug Discov. Today.

[24] Cheng E., Hung S.C., Lin T.Y. (2025). Association of trimethylamine n-oxide and metabolites with kidney function decline in patients with chronic kidney disease. Clin. Nutr..

[25] Vitetta L., Linnane A.W., Gobe G.C. (2013). From the gastrointestinal tract (GIT) to the kidneys: Live bacterial cultures (probiotics) mediating reductions of uremic toxin levels via free radical signaling. Toxins.

[26] Graboski A.L., Redinbo M.R. (2020). Gut-derived protein-bound uremic toxins. Toxins.

[27] Vaziri N.D., Wong J., Pahl M., Piceno Y.M., Yuan J., Desantis T.Z., Ni Z., Nguyen T.-H., Andersen G.L. (2013). Chronic kidney disease alters intestinal microbial flora. Kidney Int..

[28] Ramezani A., Raj D.S. (2013). The gut microbiome, kidney disease, and targeted interventions. J. Am. Soc. Nephrol..

[29] Vaziri N.D. (2012). CKD impairs barrier function and alters microbial flora of the intestine: A major link to inflammation and uremic toxicity. Curr. Opin. Nephrol. Hypertens..

[30] Barrios C., Beaumont M., Pallister T., Villar J., Goodrich J.K., Clark A., Pascual J., Ley R.E., Spector T.D., Bell J.T. (2015). Gut-microbiota-metabolite axis in early renal function decline. PLoS ONE.

[31] Fukuuchi F., Hida M., Aiba Y., Koga Y., Endoh M., Kurokawa K., Sakai H. (2002). Intestinal bacteria-derived putrefactants in chronic renal failure. Clin. Exp. Nephrol..

[32] Strid H., Simrén M., Stotzer P.O., Ringström G., Abrahamsson H., Björnsson E.S. (2003). Patients with chronic renal failure have abnormal small intestinal motility and a high prevalence of small intestinal bacterial overgrowth. Digestion.

[33] Wang F., Zhang P., Jiang H., Cheng S. (2012). Gut bacterial translocation contributes to microinflammation in experimental uremia. Dig. Dis. Sci..

[34] Wong J., Piceno Y.M., Desantis T.Z., Pahl M., Andersen G.L., Vaziri N.D. (2015). Expansion of urease- and uricase-containing, indole- and p-cresol-forming and contraction of short-chain fatty acid-producing intestinal microbiota in ESRD. Am. J. Nephrol..

[35] Peters B.A., Qi Q., Usyk M., Daviglus M.L., Cai J., Franceschini N., Lash J.P., Gellman M.D., Yu B., Boerwinkle E. (2023). Association of the gut microbiome with kidney function and damage in the hispanic community health study/study of latinos (HCHS/SOL). Gut Microbes..

[36] Gargiulo S., Barone V., Bonente D., Tamborrino T., Inzalaco G., Gherardini L., Bertelli E., Chiariello M. (2024). Integrated ultrasound characterization of the diet-induced obesity (DIO) model in young adult c57bl/6j mice: Assessment of cardiovascular, renal and hepatic changes. J. Imaging..

[37] Liu Y.-J., Wang Y., Xu L.-X., Yang J., Zhao Y., Qiao J., Li N., Li Y., Lv D.-Q., Sun W.-Y. (2023). Relationship between dietary patterns and diabetic microvascular complications in patients with type 2 diabetes mellitus. Eur. Rev. Med. Pharmacol. Sci..

[38] Salminen S., Collado M.C., Endo A., Hill C., Lebeer S., Quigley E.M., Sanders M.E., Shamir R., Swann J.R., Szajewska H. (2021). The international scientific association of probiotics and prebiotics (ISAPP) consensus statement on the definition and scope of postbiotics. Nat. Rev. Gastroenterol. Hepatol..

[39] Cornall L.M., Mathai M.L., Hryciw D.H., Mcainch A.J. (2013). The therapeutic potential of GPR43: A novel role in modulating metabolic health. Cell Mol. Life Sci..

[40] Jiao A., Zhao Y., Chu L., Yang Y., Jin Z. (2024). A review on animal and plant proteins in regulating diabetic kidney disease: Mechanism of action and future perspectives. J. Funct. Foods.

[41] Guida B., Germanò R., Trio R., Russo D., Memoli B., Grumetto L., Barbato F., Cataldi M. (2014). Effect of short-term synbiotic treatment on plasma p-cresol levels in patients with chronic renal failure: A randomized clinical trial. Nutr. Metab. Cardiovasc. Dis..

[42] Poesen R., Evenepoel P., de Loor H., Delcour J.A., Courtin C.M., Kuypers D., Augustijns P., Verbeke K., Meijers B. (2016). The influence of prebiotic arabinoxylan oligosaccharides on microbiota derived uremic retention solutes in patients with chronic kidney disease: A randomized controlled trial. PLoS ONE.

[43] Meijers B.K., de Preter V., Verbeke K., Vanrenterghem Y., Evenepoel P. (2010). P-cresyl sulfate serum concentrations in haemodialysis patients are reduced by the prebiotic oligofructose-enriched inulin. Nephrol. Dial. Transplant..

[44] Esgalhado M., Kemp J.A., Azevedo R., Paiva B.R., Stockler-Pinto M.B., Dolenga C.J., Borges N.A., Nakao L.S., Mafra D. (2018). Could resistant starch supplementation improve inflammatory and oxidative stress biomarkers and uremic toxins levels in hemodialysis patients? A pilot randomized controlled trial. Food Funct..

[45] Sirich T.L., Plummer N.S., Gardner C.D., Hostetter T.H., Meyer T.W. (2014). Effect of increasing dietary fiber on plasma levels of colon-derived solutes in hemodialysis patients. Clin. J. Am. Soc. Nephrol..

[46] Kieffer D.A., Piccolo B.D., Vaziri N.D., Liu S., Lau W.L., Khazaeli M., Nazertehrani S., Moore M.E., Marco M.L., Martin R.J. (2016). Resistant starch alters gut microbiome and metabolomic profiles concurrent with amelioration of chronic kidney disease in rats. Am. J. Physiol. Ren. Physiol..

[47] Salarolli R.T., Alvarenga L., Cardozo L.F.M.F., Teixeira K.T.R., Moreira L.d.S.G., Lima J.D., Rodrigues S.D., Nakao L.S., Fouque D., Mafra D. (2021). Can curcumin supplementation reduce plasma levels of gut-derived uremic toxins in hemodialysis patients? A pilot randomized, double-blind, controlled study. Int. Urol. Nephrol..

[48] Annunziata G., Maisto M., Schisano C., Ciampaglia R., Narciso V., Tenore G.C., Novellino E. (2019). Effects of grape pomace polyphenolic extract (taurisolo^®^) in reducing TMAO serum levels in humans: Preliminary results from a randomized, placebo-controlled, cross-over study. Nutrients.

[49] Tenore G.C., Caruso D., Buonomo G., D’avino M., Ciampaglia R., Maisto M., Schisano C., Bocchino B., Novellino E. (2019). Lactofermented annurca apple puree as a functional food indicated for the control of plasma lipid and oxidative amine levels: Results from a randomised clinical trial. Nutrients.

[50] Wang Z., Peters B.A., Yu B., Grove M.L., Wang T., Xue X., Thyagarajan B., Daviglus M.L., Boerwinkle E., Hu G. (2024). Gut microbiota and blood metabolites related to fiber intake and type 2 diabetes. Circ. Res..

[51] Conlon M.A., Kerr C.A., Mcsweeney C.S., Dunne R.A., Shaw J.M., Kang S., Bird A.R., Morell M.K., Lockett T.J., Molloy P.L. (2012). Odporne skrobie chronią przed uszkodzeniem kolonicznego DNA i zmieniają mikroflorę i ekspresję genów u szczurów karmionych zachodnią dietą. J. Nutr..

[52] Snelson M., Kellow N.J., Coughlan M.T. (2019). Modulation of the gut microbiota by resistant starch as a treatment of chronic kidney diseases: Evidence of efficacy and mechanistic insights. Adv. Nutr..

[53] Szczuko M., Kikut J., Maciejewska D., Kulpa D., Celewicz Z., Ziętek M. (2020). The associations of SCFA with anthropometric parameters and carbohydrate metabolism in pregnant women. Int. J. Mol. Sci..

[54] Shamloo M., Mollard R., Wang H., Kingra K., Tangri N., Mackay D. (2022). A randomized double-blind cross-over trial to study the effects of resistant starch prebiotic in chronic kidney disease (respeckd). Trials.

[55] Metzler-zebeli B.U., Canibe N., Montagne L., Freire J., Bosi P., Prates J.A.M., Tanghe S., Trevisi P. (2019). Resistant starch reduces large intestinal ph and promotes fecal lactobacilli and bifidobacteria in pigs. Animal.

[56] Sybille T., June Z., Michael K., Roy M., Maria L.M. (2013). The intestinal microbiota in aged mice is modulated by dietary resistant starch and correlated with improvements in host responses. FEMS Microbiol. Ecol..

[57] Tian E., Wang F., Zhao L., Sun Y., Yang J. (2023). The pathogenic role of intestinal flora metabolites in diabetic nephropathy. Front Physiol..

[58] Tao S., Li L., Li L., Liu Y., Ren Q., Shi M., Liu J., Jiang J., Ma H., Huang Z. (2019). Understanding the gut–kidney axis among biopsy-proven diabetic nephropathy, type 2 diabetes mellitus and healthy controls: An analysis of the gut microbiota composition. Acta Diabetol..

[59] Martínez I., Kim J., Duffy P.R., Schlegel V.L., Walter J. (2010). Resistant starches types 2 and 4 have differential effects on the composition of the fecal microbiota in human subjects. PLoS ONE.

[60] Baxter N.T., Schmidt A.W., Venkataraman A., Kim K.S., Waldron C., Schmidt T.M., Blaser M.J., Britton R., Walter J. (2019). Dynamics of human gut microbiota and short-chain fatty acids in response to dietary interventions with three fermentable fibers. Mbio.

[61] Venkataraman A., Sieber J.R., Schmidt A.W., Waldron C., Theis K.R., Schmidt T.M. (2016). Variable responses of human microbiomes to dietary supplementation with resistant starch. Microbiome.

[62] Alfa M.J., Strang D., Tappia P.S., Graham M., van Domselaar G., Forbes J.D., Laminman V., Olson N., Degagne P., Bray D. (2018). A randomized trial to determine the impact of a digestion resistant starch composition on the gut microbiome in older and mid-age adults. Clin. Nutr..

[63] Walker A.W., Ince J., Duncan S.H., Webster L.M., Holtrop G., Ze X., Brown D., Stares M.D., Scott P., Bergerat A. (2011). Dominant and diet-responsive groups of bacteria within the human colonic microbiota. ISME J..

[64] Drake A.M., Coughlan M.T., Christophersen C.T., Snelson M. (2022). Resistant starch as a dietary intervention to limit the progression of diabetic kidney disease. Nutrients.

[65] Jia X.H., Wang S.Y., Sun A.Q. (2024). Dietary fiber intake and its association with diabetic kidney disease in american adults with diabetes: A cross-sectional study. World J. Diabetes.

[66] Pengrattanachot N., Thongnak L., Lungkaphin A. (2022). The impact of prebiotic fructooligosaccharides on gut dysbiosis and inflammation in obesity and diabetes related kidney disease. Food Funct..

[67] Costabile A., Kolida S., Klinder A., Gietl E., Bauerlein M., Frohburg C., Landschutze V., Gibson G.R. (2010). A double-blind, placebo-controlled, cross-over study to establish the bifidogenic effect of a very-long chain inulin extracted from globe artichoke (cynara scolymus) in healthy subjects. Br. J. Nutr..

[68] Ramnani P., Gaudier E., Bingham M., van Bruggen P., Tuohy K.M., Gibson G.R. (2010). Prebiotic effect of fruit and vegetable shots containing jerusalem artichoke inulin: A human intervention study. Br. J. Nutr..

[69] Calame W., Weseler A.R., Viebke C., Flynn C., Siemensma A.D. (2008). Gum arabic establishes prebiotic functionality in healthy human volunteers in a dose-dependent manner. Br. J. Nutr..

[70] Mirmiran P., Houshialsadat Z., Gaeini Z., Bahadoran Z., Azizi F. (2020). Functional properties of beetroot (beta vulgaris) in management of cardio-metabolic diseases. Nutr. Metab..

[71] Jie Z., Bang-yao J., Ming-jie X., Hai-wei L., Zu-kang Z., Ting-song W., Craig S.A.S. (2000). Studies on the effects of polydextrose intake on physiologic functions in chinese people. Am. J. Clin. Nutr..

[72] Hengst C., Ptok S., Roessler A., Fechner A., Jahreis G. (2009). Effects of polydextrose supplementation on different faecal parameters in healthy volunteers. Int. J. Food Sci. Nutr..

[73] Lefranc-millot C., Gruerin-deremaux L., Wils D., Neut C., Miller L.E., Saniez-degrave M.H. (2012). Impact of a resistant dextrin on intestinal ecology: How altering the digestive ecosystem with NUTRIOSE, a soluble fiber with prebiotic properties, may be beneficial for health. J. Int. Med. Res..

[74] Chen H., Nie Q., Hu J., Huang X., Zhang K., Nie S. (2019). Glucomannans alleviated the progression of diabetic kidney disease by improving kidney metabolic disturbance. Mol. Nutr. Food Res..

[75] Cheng X., Zhou T., He Y., Xie Y., Xu Y., Huang W. (2022). The role and mechanism of butyrate in the prevention and treatment of diabetic kidney disease. Front. Microbiol..

[76] Mafra D., Kemp J.A., Borges N.A., Wong M., Stenvinkel P. (2023). Gut microbiota interventions to retain residual kidney function. Toxins.

